# Effect of Exposure to Seminal Plasma Through Natural Mating in Cattle on Conceptus Length and Gene Expression

**DOI:** 10.3389/fcell.2020.00341

**Published:** 2020-05-12

**Authors:** Yentel Mateo-Otero, José María Sánchez, Sandra Recuero, Sandra Bagés-Arnal, Michael McDonald, David A. Kenny, Marc Yeste, Pat Lonergan, Beatriz Fernandez-Fuertes

**Affiliations:** ^1^Biotechnology of Animal and Human Reproduction (TechnoSperm), Institute of Food and Agricultural Technology, University of Girona, Girona, Spain; ^2^Unit of Cell Biology, Department of Biology, Faculty of Sciences, University of Girona, Girona, Spain; ^3^School of Agriculture and Food Science, University College Dublin, Dublin, Ireland; ^4^Animal and Bioscience Research Department, Animal and Grassland Research and Innovation Centre, Teagasc Grange, Dunsany, Ireland

**Keywords:** seminal plasma, embryo development, corpus luteum, cattle, gene expression

## Abstract

A growing body of evidence suggests that paternal factors have an impact on offspring development. These studies have been mainly carried out in mice, where seminal plasma (SP) has been shown to regulate endometrial gene expression and impact embryo development and subsequent offspring health. In cattle, infusion of SP into the uterus also induces changes in endometrial gene expression, however, evidence for an effect of SP on early embryo development is lacking. In addition, during natural mating, the bull ejaculates in the vagina; hence, it is not clear whether any SP reaches the uterus in this species. Thus, the aim of the present study was to determine whether SP exposure leads to improved early embryo survival and developmental rates in cattle. To this end, Day 7 *in vitro* produced blastocysts were transferred to heifers (12–15 per heifer) previously mated to vasectomized bulls (*n* = 13 heifers) or left unmated (*n* = 12 heifers; control). At Day 14, heifers were slaughtered, and conceptuses were recovered to assess size, morphology and expression of candidate genes involved in different developmental pathways. Additionally, CL volume at Day 7, and weight and volume of CL at Day 14 were recorded. No effect of SP on CL volume and weight not on conceptus recovery rate was observed. However, filamentous conceptuses recovered from SP-exposed heifers were longer in comparison to the control group and differed in expression of *CALM1*, *CITED1*, *DLD*, *HNRNPDL*, *PTGS2*, and *TGFB3*. In conclusion, data indicate that female exposure to SP during natural mating can affect conceptus development in cattle. This is probably achieved through modulation of the female reproductive environment at the time of mating.

## Introduction

Despite the molecular complexity underlying the critical processes that take place in the peri-conception and early preimplantation period, the success of *in vitro* reproductive techniques suggests that the requirements of the embryo can be met by a relative simple set of media, and that embryonic development, at least to the blastocyst stage, occurs independently of interaction with the female reproductive tract. While blastocyst stage embryos can induce changes in the endometrium (Sponchiado et al., 2017; Passaro et al., 2018, 2019) as well as the uterine lumen metabolite composition (Sponchiado et al., 2019), mainly through embryo-derived interferon-tau (Passaro et al., 2019), whether this interaction plays an important role in embryo survival is questionable given the fact that embryos can be transferred to a virgin uterus as late as Day 16 and still establish a pregnancy (Betteridge et al., 1980). Nonetheless, it is becoming apparent that offspring health can be affected by the environmental conditions experienced during gamete maturation, embryo development and fetal growth (Hanson and Gluckman, 2014). While the link between maternal environment and embryo and offspring wellbeing has been investigated in detail, the role of paternal factors in directing offspring development has been somewhat overlooked (Morgan and Watkins, 2019). However, there is growing evidence that paternal nutrition and body mass composition have direct impact on DNA integrity, sperm quality and epigenetic status (Fleming et al., 2018), which has an effect on the metabolic function of the offspring in mice (Bromfield et al., 2014; Watkins and Sinclair, 2014; Watkins et al., 2017, 2018). In addition, seminal plasma (SP) has been shown to modulate gene expression and the immune response of the female reproductive tract in some species such as mice, human and cattle (Fazeli et al., 2004; Sharkey et al., 2007; Chen et al., 2014; Ibrahim et al., 2019).

Seminal plasma, a fluid resulting primarily from the secretions of the male accessory glands, transports, nourishes and protects sperm at the time of ejaculation (Bromfield, 2016). At this time, sperm are coated by SP proteins that are believed to prevent capacitation until they are close to the oocyte, and that modify sperm metabolism and motility (Vicens et al., 2014).

Female exposure to SP has been shown to improve embryo development and survival in mice (Bromfield et al., 2014), humans (Crawford et al., 2015), pigs (O’Leary et al., 2004) and golden hamsters (O et al., 1988). In addition, in llamas and rabbits, species with induced ovulation, SP has been reported to stimulate ovulation through nerve growth factor (Silva et al., 2011; Ratto et al., 2012, 2019; Adams and Ratto, 2013). It is thought that the beneficial effect of SP on the embryo is due in part to the immunoregulatory role. Indeed, leukocyte infiltration in response to SP in mice (Hunt and Robertson, 1996; Tremellen et al., 1998) and pigs (O’Leary et al., 2004) has been observed. Traditionally, this migration of immune cells was thought to solely serve the purpose of clearing microorganisms and excess sperm (O’Leary et al., 2004). However, it has been demonstrated that seminal Transforming Growth Factor Beta (TGFβ) stimulates Granulocyte-Macrophage Colony-Stimulating Factor (GM-CSF) secretion by uterine epithelial cells *in vivo* and *in vitro* in mice (Robertson et al., 1997; Tremellen et al., 1998; Moldenhauer et al., 2010). This factor promotes a pro-inflammatory cytokine and chemokine cascade, which recruits immune cells into the endometrial lumen and induces differentiation of tolerogenic dendritic cells and regulatory T cells (Treg cells) (Robertson et al., 2013). This differentiation depends on the micro-environmental cytokine signals which control the transition of naïve Th0 cells into Treg cells. As a result, immune tolerance to paternal antigens is probably established, which improves the ability of the semi-allogenic embryo to implant and develop normally (Robertson et al., 2009, 2018; Guerin et al., 2011). In addition to facilitating embryo implantation, in the mouse, exposure to semen induces the oviductal and uterine synthesis of embryotrophic cytokines such as GM-CSF, Interleukin 6 (IL6) and Leukaemia Inhibitory Factor (LIF) (Robertson, 2007). In this species, these factors have been shown to improve fetal growth and placentation (Sjöblom et al., 2005). Finally, regulation of ovarian function by SP has also been reported. In mice, the macrophage population in the corpus luteum (CL) has been observed to increase one day after mating in response to uterine exposure to SP (Gangnuss et al., 2004). Similarly, an increase in CL size and progesterone (P4) concentration in peripheral blood after SP exposure in pigs has been reported (O’Leary et al., 2006). Elevated P4 during the preimplantation stage has been shown to positively influence embryo growth in mice (Aisemberg et al., 2013), ruminants (Kleemann et al., 1994; Inskeep, 2004; Clemente et al., 2009; O’Hara et al., 2014a), alpacas (Bravo and Diaz, 2010), and pigs (Ashworth, 1991; Jindal et al., 1997). This is, at least in part, due to P4-stimulated endometrial secretions, collectively termed histotroph, which support conceptus development, implantation, and placentation (Simintiras et al., 2019). Thus, the SP-induced increase in P4 likely benefits embryo development and subsequent survival.

Despite the extensive body of evidence in the mouse, in cattle, the effect of SP on embryo development and survival is not clear. Both sperm and SP have been shown to induce expression of pro-inflammatory-related genes in the endometrium after infusion of SP into the bovine uterus (Elweza et al., 2018; Ibrahim et al., 2019; Ortiz et al., 2019). However, using a similar infusion technique, no improvement in the fertility of dairy heifers (Odhiambo et al., 2009) or cows (Ortiz et al., 2019) was observed. These apparent inter-species differences in the role of SP in fertility may be due, in part, to the known variation in composition of this fluid (Rodger, 1976; Druart et al., 2013), owing to differences in accessory gland size, type and level of fluid contribution to the ejaculate (Bedford, 2015). Indeed, caution is needed when interpreting some of the aforementioned bovine studies as some used SP collected by electroejaculation (Ibrahim et al., 2019), others by artificial vagina (Odhiambo et al., 2009) and others do not mention the collection method (Elweza et al., 2018; Ortiz et al., 2019). Collection method significantly influences the composition of SP (Rego et al., 2015) and consequently has a tremendous impact on endometrial response *in vitro* (Fernandez-Fuertes et al., 2019). As a result of these issues, interpretation of the available data in cattle is challenging.

In addition, the site of ejaculate deposition (i.e., intravaginal or intrauterine) likely determines differences in the response of female reproductive tissues to SP. Due to the characteristics of mating in rodents (Dean et al., 2011) and pigs (Hunter, 1981), SP comes into direct contact with the uterus. In contrast, during natural mating, the bull deposits the ejaculate in the anterior vagina of the cow (Hawk, 1983). Thus, it is not clear if any SP reaches the uterus in this species. However, at ejaculation, SP proteins bind to the sperm membrane (Pini et al., 2016), and can therefore be carried by these cells into more distal regions of the female reproductive tract. In fact, some SP proteins that bind to sperm, such as Binder of Sperm Protein (Suarez and Pacey, 2006) and osteopontin (Souza et al., 2008), have previously been described to influence embryo development *in vitro* in pigs (Hao et al., 2008) and cattle (Gonçalves et al., 2008; Monaco et al., 2009; Rodríguez-Villamil et al., 2016). However, although bovine sperm can bind to both endometrial and oviductal cells *in vitro* and stimulate mRNA expression of different cytokines (Yousef et al., 2016; Elweza et al., 2018; Ezz et al., 2019), incubation of endometrial explants with cauda epididymis sperm (which are mature but have had no contact with SP) or Percoll-washed ejaculated sperm did not induce differences in mRNA expression of some of those cytokines (Fernandez-Fuertes et al., 2019). Thus, it is not clear if SP-derived proteins, rather than intrinsic sperm proteins, are responsible for eliciting changes in the endometrium of cattle.

It is also possible that, in species that deposit semen in the vagina, SP elicits a local response that propagates to more distal regions of the female reproductive tract. In humans, unprotected vaginal coitus leads to enhanced cytokine and chemokine expression in the cervix (Sharkey et al., 2012). These factors could travel through local circulation to elicit changes in other reproductive organs, as seems to be the case in pigs, rodents, and cattle. In the bovine, SP infusion into the vagina, but not the uterus, induces an increase in endometrial epidermal growth factor concentrations (Badrakh et al., 2020). Whereas mating (in the mouse) or infusion of SP (in the sow) have been shown to have an effect on ovarian function (Gangnuss et al., 2004; O’Leary et al., 2006).

Taken collectively, the literature suggests that although SP is not essential for pregnancy success, it can improve embryo development and survival through modulation of the maternal environment. However, to the best of our knowledge, there is currently no evidence for a role of SP in early embryo development in cattle *in vivo*. Thus, the aim of the present study was to assess the effect of SP exposure in cattle through natural mating on pre-implantation embryo survival and conceptus development. To this end, a model in which heifers were mated to vasectomized bulls (which only ejaculate SP) or left unmated (control) was used. *In vitro* produced embryos were transferred to the heifers at Day 7 post-mating and were recovered after slaughter at Day 14 to assess conceptus size, morphology and gene expression. In addition, CL volume at Day 7, as well as weight and volume at Day 14 were analyzed.

## Materials and Methods

### Animals

All experimental procedures involving animals were approved by the Animal Research Ethics Committee of University College Dublin, Ireland, and the Universitat de Girona, Spain, and licensed by the Health Products Regulatory Authority (HPRA), Ireland, in accordance with Statutory Instrument No. 543 of 2012 (under Directive 2010/63/EU on the Protection of Animals used for Scientific Purposes). Throughout the course of the experiment, all animals were housed at Teagasc Grange, Animal and Grassland Research Centre, Dunsany, Ireland.

### Experimental Design

**FIGURE 1:**
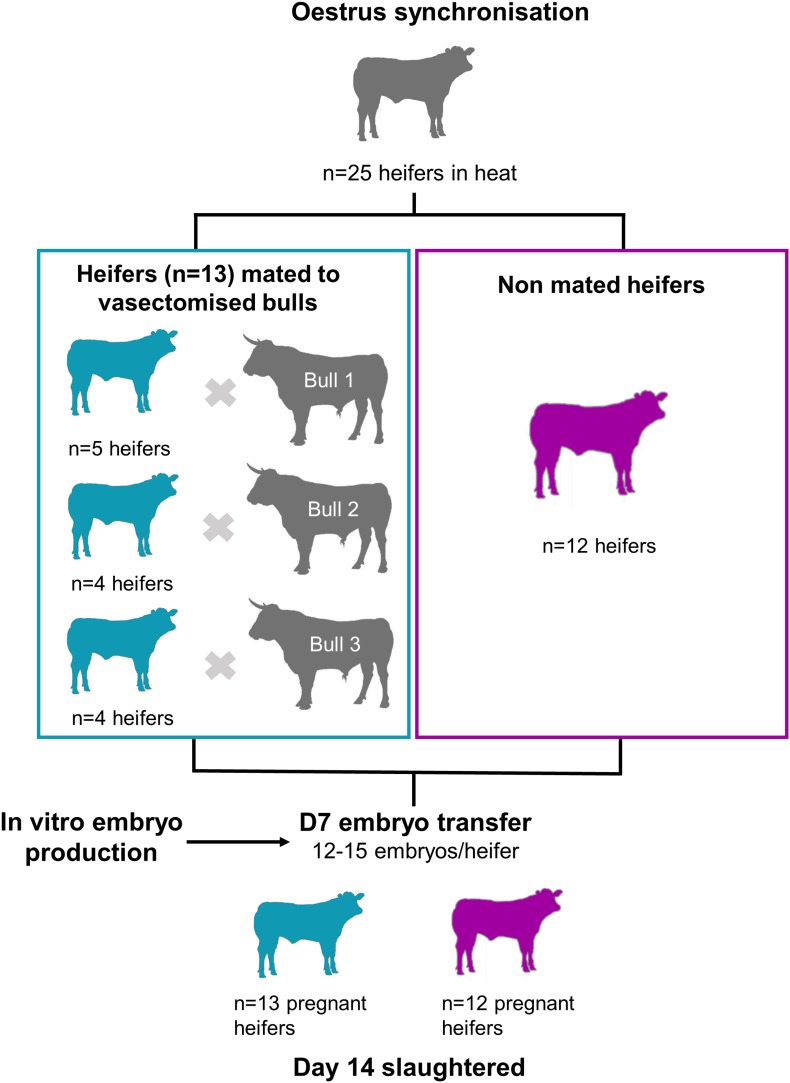
Experimental design. Heifers observed in standing estrus (*n* = 25) were blocked by weight and randomly allocated to one of two treatments: mated with vasectomised bulls (blue) or left un-mated (purple). Seven days later, 12–15 *in vitro* produced blastocysts were transferred to all heifers. Heifers were slaughtered at Day 14, and the conceptuses were recovered from the uterine horns.

The estrous cycles of crossbred beef heifers (mainly Angus and Holstein-Friesian cross; *n* = 27) were synchronized using an 8-day intravaginal device (PRID^®^ Delta, 1.55 g progesterone; Ceva Santé Animale; Libourne, France), together with a 2 mL intramuscular injection of a synthetic gonadotrophin releasing hormone (Ovarelin^®^, equivalent to 100 μg Gonadorelin; Ceva Santé Animale) administered on the day of PRID insertion. One day prior to PRID removal, all heifers received a 5 mL intramuscular injection of PGF2α (Enzaprost^®^, equivalent to 25 mg of Dinoprost; Ceva Santé Animale) to induce luteolysis. Only heifers observed in standing estrus (Day -1; *n* = 25) were blocked by weight and randomly allocated to one of two treatments: (1) mated with a vasectomized bull (*n* = 13), or (2) left unmated (control; *n* = 12). Each mated heifer was hand-mated once to one of three vasectomized Holstein Friesian bulls within 7 h of the start of standing estrus. Bulls were allowed to mate no more than twice per day and the experiment was carried out over three consecutive days. Seven days after mating, *in vitro* produced blastocysts were transferred to each heifer (*n* = 12–15 per heifer). All heifers were slaughtered in a commercial abattoir 7 days after embryo transfer to recover Day 14 conceptuses. In addition, CL volume at Day 7, and CL weight and volume at Day 14 were recorded. The experimental design is summarized in Figure 1.

### *In vitro* Embryo Production

Ovaries from cows and heifers were collected at a commercial abattoir and surface visible follicles (>2 mm) were aspirated to recover cumulus-oocyte complexes (COCs). Good quality COCs were matured in TCM-199 (Sigma Aldrich, Saint Louis, MO, United States) supplemented with 10% (v/v) FCS (Sigma Aldrich) and 10 ng/mL Epidermal Growth Factor (Merck; Darmstadt, Germany) (*n* = 50 COCs per well) for 24 h at 39°C under an atmosphere of 5% CO_2_ in air with maximum humidity. Matured COCs were fertilized using sperm from a bull of proven *in vitro* fertility at a concentration of 1 × 10^6^ sperm/mL. Frozen-thawed semen from the same bull was used throughout. Motile sperm were selected by centrifugation through a 95–45% discontinuous Percoll gradient (Merck) for 10 min at 700 g, followed by a second centrifugation in HEPES-buffered Tyrode medium (Boston BioProducts; MA, United States) at 100 g for 5 min. Gametes were co-incubated at 39°C in an atmosphere of 5% CO_2_ in air with maximum humidity. Approximately 20 h post-insemination, cumulus cells were removed, and presumptive zygotes were cultured in 25 μL droplets of synthetic oviduct fluid supplemented with 5% FCS (*n* = 25 per well) under mineral oil (Sigma Aldrich) until Day 7 (Day 0 = day of fertilization). Culture dishes were kept at 39°C under an atmosphere of 5% CO_2_ and 5% O_2_ in air with maximum humidity until Day 7. Blastocysts classified as excellent or good (following guidelines of the International Embryo Technology Society, 2009) were pooled, and then randomly loaded into straws (*n* = 12–15 embryos/straw) for embryo transfer.

### Embryo Transfer and Recovery

On Day 7 (Day 0 = day of *in vitro* fertilization for the embryos, and day after mating for the heifers) the Day 7 *in vitro*-produced blastocysts were transferred (*n* = 12–15 blastocysts/heifer) to the horn ipsilateral to the ovary bearing the CL. All heifers were slaughtered on Day 14 (7 days after embryo transfer). Reproductive tracts were recovered, gently dissected, and flushed with PBS containing 5% FCS within 30 min of slaughter. The number and dimensions (length and width) of recovered Day 14 embryos were recorded. Conceptuses were classified based on morphology as ovoid (1–4 mm), tubular (5–19 mm), or filamentous (>20 mm), based on previous studies (Ribeiro et al., 2016). Due to the large range in conceptus length in the filamentous group, the filamentous group was subdivided in short filamentous (20–25 mm) and long filamentous (>25 mm) for gene expression analysis.

### Calculation of CL Weight and Volume

Corpus luteum volume was calculated as described previously (Scully et al., 2014; Parr et al., 2017). Briefly, for Day 7 CL, the formula for the volume of a sphere was used (*V* = 4/3πr^3^). The radius was calculated as the average of the two cross-sectional ultrasound measurements (the CL diameter) divided by two. For those CL with a cavity, the volume of the cavity was calculated using the same formula and subtracted from the total CL volume.

Because measurements in three axes (a, b, c) could be taken from post-mortem Day 14 CL, the formula for the volume of an ellipsoid (*V* = 4/3πabc) was used (Grygar et al., 1997). As above, for CL with a cavity, the volume of the cavity was subtracted from the total CL volume. Moreover, the weight of luteal tissue of these CL was also recorded.

### Quantitative Real-Time PCR Analysis

Quantitative real-time PCR (RT-qPCR) was used to investigate changes in relative abundance of candidate transcripts in all Day 14 conceptuses due to treatment. A panel of five genes was used to determine conceptus gastrulation stage based on Degrelle et al. (2011): Calmodulin 1 (*CALM1*), Cbp/P300 interacting transactivator with Glu/Asp rich carboxy-terminal (*CITED1*), Dihydrolipoamide Dehydrogenase (*DLD*), Heterogeneous Nuclear Ribonucleoprotein D Like (*HNRNPDL*), and Transforming Growth Factor Beta 3 (*TGFB3*). However, under the conditions of the current study, the gene expression patterns that the authors described to classify gastrulation stage were not observed. Because the aforementioned genes are involved in different functional pathways within the conceptus, additional genes that participate in such pathways, and that have been found to be differentially expressed along development (Mamo et al., 2011; Barnwell et al., 2016) were interrogated: Caspase 3 (*CASP3*), Furin (*FURIN*), Glutathione S-Transferase Mu 1 (*GSTM1*), *IL6*, MHC Class I JSP 1 (*JSP1*), and Prostaglandin-Endoperoxide Synthase 2 (*PTGS2*).

Total RNA was extracted from entire conceptuses using Trizol reagent (Invitrogen; Carlsbad, CA, United States) and trimethylene chlorobromide (Sigma Aldrich). On-column DNase digestion and RNA clean-up was performed using the RNeasy Mini Kit (Qiagen; Hilden, Germany) following the manufacturer’s instructions. The quantity and purity of RNA was determined using the Epoch Microplate Spectrophotometer (BioTek; Winooski, VT, United States). For each sample, cDNA was prepared from 14.7 ng of total RNA (based on the sample with lowest RNA concentration) using the High-Capacity cDNA Reverse Transcription Kit (ThermoFisher Scientific; Waltham, MA, United States) according to the manufacturer’s instructions. For the PCR negative control, a retrotranscription mastermix without the enzyme was applied to an RNA pool of a representative sample of conceptuses.

All primers were designed using Primer Blast software^1^ (Table 1). In order to identify the most suitable housekeeping genes, duplicate qPCR assays were performed in a total volume of 20 μL, containing 10 μL Fast SYBR Green Master Mix (ThermoFisher Scientific), 1.2 μL forward and reverse primer mix (5 nM final concentration), 5.1 μL Nuclease-Free Water (ThermoFisher Scientific) and 2.5 ng of a representative sample of embryos. The Applied Biosystems 7500 Real-Time PCR Systems (ThermoFisher Scientific) was used and the thermo-cycling conditions were as follows: 1 cycle of holding stage at 50°C for 2 min and 95°C for 10 min; 40 cycles of cycling stage at 95°C for 15 s and 60°C for 1 min and, finally, 1 cycle of melt curve stage at 95°C for 15 s, 60°C for 1 min, 95°C for 30 s and 60°C for 15 s. The presence of a single sharp peak in the melt curve analysis confirmed the specificity of all targets. A total of eight potential reference genes [Glyceraldehyde 3-Phosphate Dehydrogenase (*GAPDH*), Actin Cytoplasmic 1 (*ACTB*), 60S Ribosomal Protein L18 (*RPL18*), Peptidyl-Prolyl *Cis*-*Trans* Isomerase A (*PPIA*), 14-3-3 Protein Zeta/Delta (*YWHAZ*), RING Finger Protein 11 (*RNF11*), Histone H3.3 (*H3F3A*), Succinate Dehydrogenase Complex Subunit A Flavoprotein Variant (*SDHA*)] were analyzed using the geNorm function of the Qbase + package (Biogazelle; Zwijnaarde, Belgium) to identify the most appropriate for the study (Vandesompele et al., 2002). Because they were more stably expressed (average GeNorm M ≤ 0.5), the reference genes selected were *RPL19* and *PPIA*.

**TABLE 1:** Primer design.

Gene	RefSeq (Bos taurus)	Forward primer	Reverse primer	Tm (°C)	Amplicon size (bp)
*RPL19*	NM_001040516.1	GAAAGGCAGGCATATGGGTA	TCATCCTCCTCATCCAGGTT	60	86
*PPIA*	NM_178320.2	CATACAGGTCCTGGCATCTTGTCC	CACGTGCTTGCCATCCAACC	60	108
*CALM1*	NM_001242572.1	GGATGGCAACGGGTACATCA	CTCCTCGTCCGTCAGCTTC	60	79
*CASP3*	NM_001077840.1	ACCAACGGACCCGTCAATTT	CCTCGGCAGGCCTGAATAAT	60	107
*CITED1*	NM_174518.1	TCACCTCCCACCAATTTATCCAA	TTGGCATTCTCCTTCACAGGT	60	110
*DLD*	NM_001206170.2	CGATGGCAGCACTCAAGTTA	CCTTGTTTTTGAAGGATACGTTG	60	306
*FURIN*	XM_024981598.1	GTTCGGCAACGTGCCCTG	TTCTTATTGGCCTCCAGGGTGAG	60	195
*GSTM1*	XM_010803234.3	GGACTTTCCCAATTTGCCCTAC	GCAATGTAGCGAAGGATGGC	60	78
*HNRNPDL*	XR_235028.4	GTGGCTATGGCGGCTATGAT	TGTTGGCCACTGTAGTCTGC	60	85
*IL6*	NM_173923.2	GCGCATGGTCGACAAAATCT	AAATCGCCTGATTGAACCCAGA	60	158
*JSP1*	XM_024983412.1	TTCCTCACCATGGGCATCATTG	ATCGTTATTCTGTTCCCGGCTG	60	172
*PTGS2*	NM_174445.2	CTGATGTTTGCATTCTTTGCCC	CTTAAGTCCACCCCATGGTTCT	60	107
*TGFB3*	NM_001101183.1	ACATAGCCAAGCAGCGGTAT	CCTAAGTTGGATTCTCTCCGCA	60	124

*RefSeq corresponds to Gene NCBI accession number. PCR conditions: melting temperature in °C (Tm) and amplicon size in bp.*

Primer efficiency was carried out for the genes of interest, and qPCR of 1:4 dilutions of a cDNA mix from a representative pool of conceptuses were analyzed. The presence of a single sharp peak in the melt curve as well as the standard curve was used to confirm primer specificity. Average primer efficiency was 93.0 ± 4.7%. The expression of these genes was individually evaluated in 76 conceptuses (20 ovoid, 20 tubular, 36 filamentous) using 2.5 ng of cDNA, 10 μL of Fast SYBR^TM^ Green Master Mix, 1.2 μL of 5 nM primers and 5.1 μL nuclease-free water, and the thermo-cycling conditions previously detailed.

The comparative Livak Ct method (ΔΔCt method; Livak and Schmittgen, 2001) was used to quantify the relative gene expression levels. First, for each conceptus, the expression of the gene of interest was normalized to the expression of the two housekeeping genes (*RPL19* and *PPIA*), using the following formula: ΔCt = Ct_gene__of interest_−Ct_RPL__19__+PPIA/__2_. To calculate the ΔΔCt, results were scaled to the average ΔCt across all conceptuses per target. The ΔCt values were used for the subsequent statistical analysis and results are presented as 2^(–Δ^ ^Δ^ ^Ct)^.

### Statistical Analysis

Data relating to conceptus and CL sizes were checked for normality and homogeneity of variance by histograms, Qplots, and formal statistical tests as part of the UNIVARIATE procedure of SAS (version 9.1.3; SAS Institute, Cary, NC, United States). Conceptus size data were not normally distributed and, as such, were transformed by raising the variable to the power of lambda. The appropriate lambda value was obtained by conducting a Box–Cox transformation analysis using the TRANSREG procedure of SAS. The transformed data were used to calculate *P* values. The corresponding least squares means and standard error of the non-transformed data are presented in the results. Conceptus data and CL data (on Days 7 and 14) were analyzed using a mixed model (PROC MIXED of SAS). The model had experimental treatment (Control or Vasectomized) as a fixed effect, and heifer within treatment was included as a random effect. Differences between treatments were determined by *F* tests using type III sums of squares. The PDIFF command incorporating the Tukey test was applied to evaluate pairwise comparisons between treatment means. Values were statistically significantly different when *P* ≤ 0.05 and considered a tendency when *P* ≤ 0.10.

Gene expression data were analyzed with IBM SPSS 25.0 for Windows (Armonk; New York, NY, United States). First, data were checked for normal distribution (Shapiro-Wilk test) and homogeneity of variance (Levene test), premises for linear models. In those cases in which these premises were not met, data (*x*) were transformed using the arcsine of the square root (arcsin √*x*). Later, data (transformed or not depending on the case) were analyzed by an ANOVA of two factors followed by a Sidak *post hoc* test for pairs comparison. Since even after arcsin-transformation, expression of *CALM1*, *CASP3*, *CITED1*, *DLD*, and *TFGB3* did not match with parametric assumptions, Scheirer-Ray-Hare and Mann-Whitney tests were used as alternatives. In all cases, the significance level was established at *P* ≤ 0.05.

## Results

### Effect of SP Exposure on CL Size

Exposure to SP through mating with a vasectomized bull did not elicit differences in the CL volume at Day 7 or at Day 14 (Day 7: 7.1 ± 0.76 cm^3^ vs. 6.5 ± 0.49 cm^3^, for mated and unmated heifers, respectively, *P* > 0.05; Day 14: 3.1 ± 0.39 cm^3^ vs. 4.5 ± 0.91 cm^3^, respectively, *P* > 0.05). Similarly, no difference in CL weight on Day 14 was observed between SP-exposed and control heifers (5.1 ± 0.46 g vs. 6.4 ± 0.79 g, respectively; *P* > 0.05).

### Effect of SP Exposure on Embryo Viability and Morphology

**FIGURE 2:**
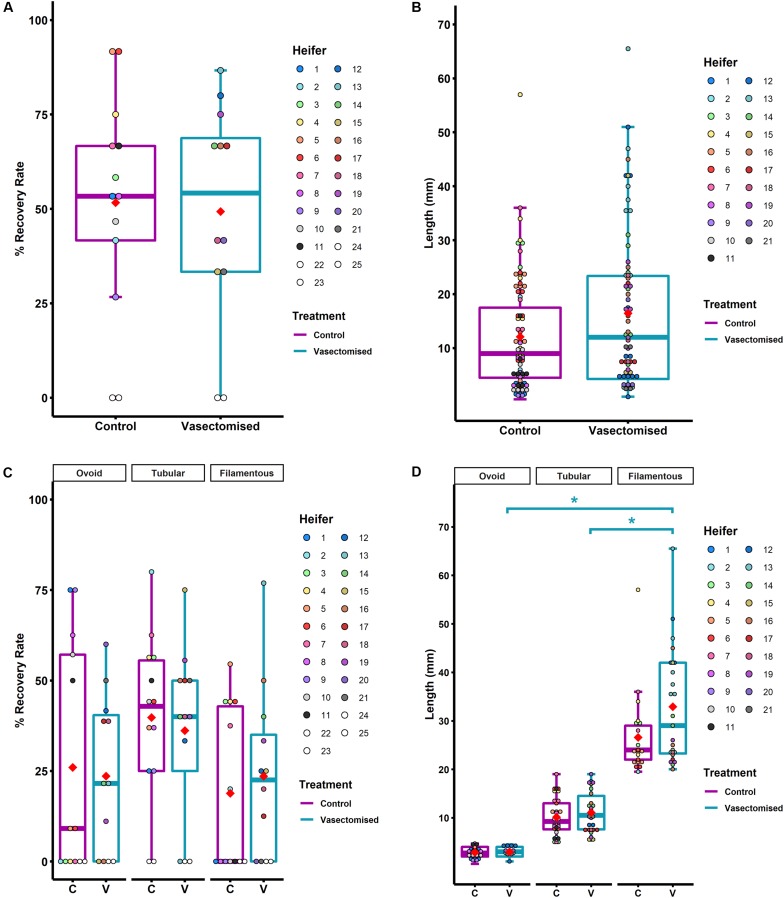
**(A)** Conceptus recovery rate at Day 14 post-mating (vasectomized; *n* = 12 heifers) or post-estrus onset (control; *n* = 13 heifers). Each color represents one heifer. **(B)** Length of the recovered conceptuses post-mating (vasectomized; *n* = 78) or post-estrus onset (control; *n* = 86). Dots represent the conceptus length and their color reflects the heifer from which they were recovered. **(C)** Conceptus recovery rate at Day 14 depending on morphology (for control (C): ovoid *n* = 24, tubular *n* = 40, filamentous *n* = 22; for vasectomized (V): ovoid *n* = 21, tubular *n* = 21, filamentous *n* = 27). Dots represent the recovery rate of each heifer for each morphology. **(D)** Length of the recovered conceptuses depending on morphology [for control (C): ovoid *n* = 24, tubular *n* = 40, filamentous *n* = 22; for vasectomized (V): ovoid *n* = 21, tubular *n* = 21, filamentous *n* = 27]. Dots represent the conceptus length and their color depends on the heifer from which they were recovered. In all graphs, means are represented as red rhombus. Control group box-plots are represented in purple and vasectomized group box-plot, in blue. For the dots, light scale colors correspond to the control group and dark scale, to heifers mated to vasectomized bulls. Differences are indicated as *P ≤ 0.05.

Conceptus recovery rate was similar from mated (exposed to SP) and unmated heifers (86/168: 51 ± 8.4% vs. 78/153: 51 ± 8.1%, respectively, *P* > 0.05; Figure 2A), indicating a lack of effect of SP-exposure on the survival of the transferred embryos. As is normal in cattle studies in which multiple-embryo transfer is carried out (O’Hara et al., 2014b), considerable variation in conceptus length within heifer was observed in both groups (CV 44–79%). However, conceptuses recovered from heifers mated to vasectomized bulls tended to be longer than those recovered from control heifers (16 ± 1.3 mm vs. 12 ± 1.2 mm, respectively, *P* = 0.07; Figure 2B).

Moreover, although no differences were observed in the percentage of ovoid, tubular and filamentous conceptuses recovered between groups (24/86: 26 ± 8.8%, 40/86: 40 ± 6.4%, 22/86: 18 ± 6.2% in control heifers and 21/78: 23 ± 6.2%, 30/78: 36 ± 6.9%, 27/78: 24 ± 6.8% in mated heifers; *P* > 0.05; Figure 2C), filamentous conceptuses recovered from SP-exposed heifers were longer than those recovered from control heifers (33 ± 2.2 mm vs. 27 ± 1.8 mm, respectively; *P* < 0.05; Figure 2D). Due to the large range in filamentous conceptus length, this group was subdivided into short filamentous (20–25 mm) and long filamentous (>25 mm) for subsequent gene expression analysis. In the control group, 6/86 conceptuses (7 ± 3.8%) were classified as long filamentous (average size 36 ± 4.7 mm; *n* = 6). In the SP-exposed group, 14/78 (18 ± 5.6%) conceptuses exhibited this morphology (average size 42 ± 2.4 mm; *n* = 14).

### Effect of SP on Conceptus Gene Expression

In order to more accurately evaluate the developmental stage of the conceptuses, the relative abundance of transcripts for five candidate genes (*CALM1*, *CITED1*, *DLD*, *HNRNPDL*, and *TFGB3)* previously described as gastrulation markers (Degrelle et al., 2011) was assessed. However, the expression profiles described by Degrelle et al. (2011) in association with different developmental stages were not observed. Nevertheless, these genes are involved in important pathways for embryo development, and their expression changes are temporally regulated. In order to better characterize these pathways, an additional set of genes related to metabolism (*GSTM1* and *PTGS2*), apoptosis (*CASP3*), development (*FURIN*), and immunology (*JSP1* and *IL6*) (Mamo et al., 2011; Barnwell et al., 2016) was also interrogated. All these genes were analyzed in individual conceptuses exhibiting different morphologies (ovoid, tubular, short filamentous, and long filamentous).

**FIGURE 3:**
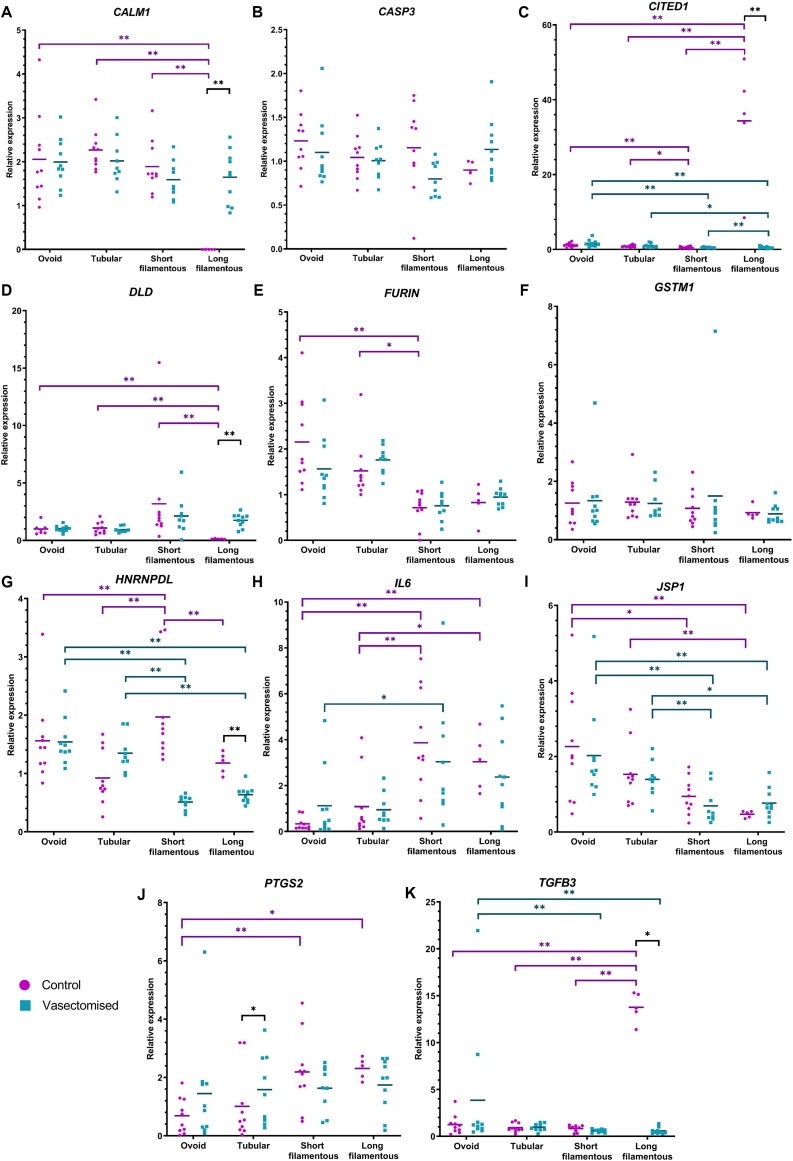
Relative expression of *CALM1* **(A)**, *CASP3* **(B)**, *CITED1* **(C)**, *DLD* **(D)**, *FURIN* **(E)**, *GSTM1* **(F)**, *HNRNPDL* **(G)**, *IL6* **(H)**, *JSP1* **(I)**, *PTGS2* **(J)**, and *TGFB3* **(K)** among the different conceptus morphologies (for control: ovoid *n* = 10, tubular *n* = 10, short filamentous *n* = 10 and long filamentous *n* = 5; for vasectomized: ovoid *n* = 10, tubular *n* = 10, short filamentous *n* = 10 and long filamentous *n* = 10). Individual expression values (calculated with 2^– ΔΔCt^ method, using the housekeeping genes *RPL19* and *PPIA*) with bar representing the mean are presented. Purple dots correspond to the control group and blue squares correspond to the vasectomized group. Differences are indicated as *P ≤ 0.05 and **P ≤ 0.01.

The relative abundance of *CITED1, HNRNPDL, IL6, JSP1*, and *TGFB3* was affected by conceptus morphology in both the control and vasectomized groups. Long and short filamentous conceptuses recovered from control or SP-exposed heifers had lower *JSP1* expression (*P* < 0.05; Figure 3I). On the other hand, in both treatments, *IL6* relative expression was higher in short filamentous conceptuses in comparison to ovoid embryos (*P* < 0.05; Figure 3H); control long and short filamentous embryos also had higher relative abundances of this gene compared to ovoid and tubular conceptuses (*P* < 0.05; Figure 3H). In control conceptuses, *CITED1* and *TGFB3* relative expression was highest in long filamentous conceptuses (*P* < 0.01; Figures 3C,K), while in the vasectomized group such conceptuses exhibited the lowest relative expression of *CITED1* (*P* < 0.05; Figure 3C) and both long and short conceptuses had lower *TGFB3* expression compared to ovoid conceptuses (*P* < 0.01; Figure 3K). Additionally, *HNRNPDL* relative expression in the control group was highest in short filamentous conceptuses (*P* < 0.01; Figure 3G), whereas in the vasectomized group, the expression of this gene was lowest in both short and long filamentous conceptuses (*P* < 0.01; Figure 3G). In addition to these, in the control group, long filamentous conceptuses also exhibited the lowest *CALM1* and *DLD* relative abundance (*P* < 0.01; Figures 3A,D); while *FURIN* relative expression in short filamentous conceptuses was lower than ovoid and tubular embryos (*P* < 0.05; Figure 3E). On the contrary, higher *PTGS2* expression was detected in long and short filamentous conceptuses in comparison to ovoid conceptuses (*P* < 0.05; Figure 3J).

Relative expression of *CALM1, CITED1, DLD, HNRNPDL, PTGS2*, and *TGFB3* differed between treatments. Long filamentous conceptuses recovered from mated heifers presented lower *CITED1, HNRNPDL*, and *TGFB3* expression, and higher *CALM1* and *DLD* expression levels, compared to morphology-matched conceptuses recovered from control heifers (*P* < 0.05; Figure 3). In addition, *PTGS2* relative expression was higher in tubular conceptuses recovered from conceptuses that developed in a SP-primed environment than in control heifers (*P* < 0.05; Figure 3J).

## Discussion

The main findings of this study are that exposure of heifers to SP through natural mating with vasectomized bulls: (1) does not elicit changes in the size of Day 7 or Day 14 CL; (2) does not improve embryo survival to Day 14, but (3) is associated with an increase in conceptus length and (4) alteration in expression of *CALM1*, *PTGS2*, *CITED1*, *DLD*, *HNRNPDL*, and *TGFB3*.

In recent years, the paternal influence on offspring health has gained increasing interest. This is due to studies showing that paternal health and nutrition can affect offspring development, and such effects can be carried over to the next generation (Morgan et al., 2020). One may immediately assume that the changes elicited in the embryo and subsequent individual are directly linked to abnormalities in the sperm of these males, which manage to fertilize the oocyte and transmit certain epigenetic signatures. However, mating of artificially-inseminated females to vasectomized mice fed different diets also has an effect on offspring outcomes (Watkins et al., 2018; Morgan et al., 2020), indicating that SP-induced changes in the female reproductive tract at the time of mating affect embryo development. Indeed, studies in mice and pigs have demonstrated that SP plays a role in the modulation of the maternal environment and, as a result, improves embryo survival and implantation (Johansson et al., 2004; O’Leary et al., 2004; Bromfield et al., 2014). In cattle, however, while SP infusion into the uterus alters the expression of certain genes (Ibrahim et al., 2019), this is not correlated with improved pregnancy rates (Odhiambo et al., 2009; Ortiz et al., 2019). These differences between species may reflect differences in SP composition (Rodger, 1976; Druart et al., 2013). As SP is a complex secretion produced by different accessory glands, variation in the type, structure and size of these organs can have a major impact on its composition (Bedford, 2015). For example, while the boar has large bulbourethral, prostate and vesicular glands, the latter two are relatively small in the bull (Druart et al., 2013). These two species only share 34% of their SP proteins in common (Druart et al., 2013). Perhaps more interesting to the subject at hand, both rodents and boars have an additional accessory gland that is lacking in the bull: the coagulating gland. This gland contributes to semen coagulation after ejaculation, which has been suggested to make sperm coating by SP proteins highly inefficient (Lefebvre et al., 2007). Thus, direct contact of the endometrium with the ejaculate might be more important in these species than in the bovine, where sperm can act as a vehicle for SP proteins. Indeed, it is important to note that in both mice and pigs, SP reaches the uterus during mating, while in cattle, the ejaculate is deposited in the anterior vagina (Hawk, 1983), and it is not clear whether any reaches the uterus without sperm involvement. Thus, models based on SP infusion directly into the uterus (Odhiambo et al., 2009; Ortiz et al., 2019) might not be representative of the events that take place physiologically. For this reason, in the current study, a model based on mating heifers to vasectomized bulls was used.

In addition to having an effect on the endometrium, studies in both mice and pigs have reported influences of SP on the ovary (Gangnuss et al., 2004; O’Leary et al., 2006). Exposure to SP (through mating in the mouse, or infusion into the uterus in the pig) led to an increase in macrophage recruitment into the ovulatory follicle (Gangnuss et al., 2004; O’Leary et al., 2006). In the pig, this was associated with an increase in CL weight at Days 5–9, which probably explains the increase in P4 secretion that was also observed at this time (O’Leary et al., 2006). Although there is inconsistent evidence on the effect of high P4 on embryo survival in pigs, with some authors reporting a positive effect (Ashworth, 1991; Jindal et al., 1997), others a negative (Mao and Foxcroft, 1998), and others a lack of effect (Muro et al., 2019), P4 prevents embryo resorption (Aisemberg et al., 2013) and is essential for timely progression of early embryogenesis (Zhang and Murphy, 2014) in mice. In cattle, elevated P4 concentrations prior to Day 7 are associated with an altered endometrial transcriptome (Forde et al., 2009) and accelerated conceptus development (Clemente et al., 2009; O’Hara et al., 2014a). Thus, increased P4 output could be one mechanism through which SP induces an increase in embryo survival in this species. However, in the present study, no differences in CL volume at Days 7 and 14, nor in Day 14 CL weight were observed between treatments.

Exposure to SP at the time of mating had no effect on embryo survival to Day 14 following transfer on Day 7. Recovery rate is related to conceptus survival, as those who die degenerate and are not recovered on Day 14. This is consistent with the studies that indicate that SP infusion into the uterus does not lead to improved pregnancy rates in cattle (Odhiambo et al., 2009; Pfeiffer et al., 2012; Ortiz et al., 2019), and contrasts with some literature available in other species where exposure to SP leads to improved embryo survival and early embryo development (Johansson et al., 2004; O’Leary et al., 2004; Bromfield et al., 2014). Despite the lack of differences regarding embryo survival, filamentous conceptuses recovered from heifers that had been mated were longer than those recovered from control heifers. As already mentioned, large variation in conceptus size recovered from the same recipient is typically observed when multiple embryo transfer is carried out (O’Hara et al., 2014b; Barnwell et al., 2016) and is also seen after insemination in single ovulating cows (Ribeiro et al., 2016). Nevertheless, when conceptuses were grouped according to morphology (thus, reducing variation), filamentous conceptuses recovered from mated heifers were longer than their control counterparts. It is not clear whether an increase in conceptus length on as given day is a positive or negative phenomenon. On the one hand, a higher number of trophectoderm cells will ultimately lead to an increase in the secretion of IFNT, the maternal recognition signal in cattle. Maternal recognition of pregnancy in this species takes place around Day 16 (Sánchez et al., 2018). At this time, conceptuses that are not able to produce sufficient amounts of IFNT will be lost due to their inability to prevent luteolysis. On the other hand, asynchronous transfer of embryos, in which a Day 7 embryo is transferred to a Day 9, results in higher conceptus length but this does not translate into higher pregnancy rates (Randi et al., 2016).

The success of embryo transfer (in the absence of exposure of the reproductive tract of the recipient to either sperm or SP) in many livestock species, where pregnancy rates are comparable to those achieved with artificial insemination (Drost et al., 1999; Sartori et al., 2006), indicates that exposure to SP is not essential for pregnancy. However, as mentioned above, this factor does seem to have an impact on embryo and offspring metabolism and overall health. Having observed differences in conceptus length between embryos developing in an environment that had been exposed to SP or not, the next aim was to determine whether this difference in size was accompanied by a difference in development stage. Because morphology and size might not be representative of the developmental stage of the conceptus, a panel of genes previously reported to be markers of gastrulation stage (Degrelle et al., 2011) was evaluated. However, in the present study, the expression profiles described by Degrelle et al. (2011) were not observed, but we did detect differences in the expression of these genes between morphologies. In both groups, filamentous conceptuses exhibited the highest expression of *IL6*, and the lowest expression of *JSP1*. This expression pattern is consistent with prior studies, who detected upregulation of *IL6* and downregulation of *JSP1* in Day 15 bovine long conceptuses (measuring 24.7 ± 1.9) in comparison to short conceptuses (measuring 4.2 ± 0.1; Barnwell et al., 2016). On the other hand, in the present study, control filamentous conceptuses exhibited the highest *CITED1* relative expression, while the opposite was true in conceptuses recovered from mated heifers. In a study by Mamo et al. (2011), Days 16 and 19 bovine conceptuses had higher expression of *CITED2* (an important paralog of *CITED1*) than Days 7, 10 and 13 conceptuses, seemingly agreeing with our control group. *HNRNPDL* and *TGFB3* relative abundance also presented different pattern between conceptuses recovered from mated or unmated animals. While control filamentous embryos had the highest relative expression of both genes, the morphology-matched conceptuses in the vasectomized group has the lowest expression.

In addition, *CALM1*, *DLD*, and *FURIN* relative expression was lowest in filamentous conceptuses, whereas *PTGS2* was highest, only in the control group. In accordance with our results, *PTGS2* expression has previously been shown to be upregulated in Day 15 long conceptuses in comparison to age-matched short conceptuses (Barnwell et al., 2016), and in Days 16 and 19 in comparison to Days 7, 10, and 13 conceptuses (Mamo et al., 2011). However, *FURIN* expression was reported to be also upregulated in Days 16 and 19 in comparison to Days 7, 10, and 13 conceptuses (Mamo et al., 2011), in disagreement to our findings.

Although the pattern described by Degrelle et al. (2011) did not allow us to determine the gastrulation stage of our conceptuses, these marker genes are involved in different pathways important to embryo survival and development. Thus, the panel of genes was supplemented with additional ones in order to describe the effect of the SP-exposed environment on conceptus apoptosis, metabolism, development, and immunology. Most of the differences between treatments detected in gene expression were observed when comparing long filamentous conceptuses. Indeed, conceptuses exhibiting this morphology had different *CALM1*, *CITED1*, *DLD*, *HNRNPDL*, *PTGS2*, and *TGFB3* expression levels depending on whether they developed in a mated or unmated heifer. Interestingly, in the control group, expression levels of these genes differed between short and long conceptuses, whereas this behavior was not detected in the conceptuses from the vasectomized group. This hints at changes in the regulation of different pathways occurring in very large embryos, which is altered by a different uterine environment elicited by SP exposure.

The apoptosis process was evaluated by analyzing *HNRNPDL* and *CASP3* relative expression. Lower levels of *HNRNPDL* relative expression were observed in long filamentous embryos from the SP-exposed group than in the control. *HNRNPDL* encodes for the heterogeneous nuclear ribonucleoprotein D Like (hnRNPDL), a RNA-binding protein which binds heterogeneous nuclear RNA (hnRNA) to regulate pre-mRNA in the nucleus (Geuens et al., 2016). When hnRNPDL binds the specific mRNA, it induces the decay of the molecule (Fialcowitz et al., 2005) and, for this reason, it is considered to repress the gene expression of its targets. One of its potential targets is the Cell Division Cycle and Apoptosis Regulator 1 (*CCAR1*) (Li et al., 2019), which acts as a key intracellular transducer for apoptosis (Rishi et al., 2003). In addition, hnRNPDL also regulates the expression of cytochrome C oxidase subunit 5B (*COX5B*), a mitochondrial energy-generating enzyme critical for the proper functioning of cells. The disruption of its expression by hnRNPDL may result in the cease of ATP generation and, therefore, the induction of apoptosis (Safavizadeh et al., 2012). Thus, a lower expression of *HNRNPDL* in the embryos could indicate lower levels of apoptosis. However, *CASP3*, a gene that codes for one of the proteases that initiates the execution pathway of apoptosis (intrinsic and the extrinsic apoptotic pathways diverge), did not differ between treatments. This suggests that SP-induced changes of *HNRNPDL* do not relate to apoptotic pathways in the conceptus and, therefore, its biological meaning remains unclear.

Conceptus metabolism was assessed by evaluating the relative expression of *DLD*, *GSTM1*, and *PTGS2*. *DLD* and *PTGS2* relative expression was higher in long filamentous or tubular embryos recovered from mated heifers than unmated heifers. *DLD* encodes for the mitochondrial dihydrolipoamide dehydrogenase, a member of the class-I pyridine nucleotide-disulfide oxidoreductase family crucial for embryo energy production (Leese, 1991; Johnson et al., 2009). Moreover, this enzyme seems to be essential for preimplantation embryos as *DLD* knockout mice embryos are unable to undergo gastrulation (Johnson et al., 1997). On the other hand, PTGS2 is the key enzyme in prostaglandin biosynthesis, which may mediate the effects of progesterone and IFNT in the endometrium and is highly expressed in the trophectoderm of ovine (Charpigny et al., 1997), bovine (Barnwell et al., 2016), porcine (Blomberg et al., 2006), and murine (Lim et al., 1999) embryos. The importance of this enzyme in embryo development is highlighted by the fact that *PTGS2* is downregulated in both *in vivo*- and *in vitro*-produced embryos that result in no pregnancy (El-Sayed et al., 2006; Ghanem et al., 2011). Considering both genes, it seems that SP may have an impact on the development in critical embryo stages such as tubular and filamentous embryos.

The relative expression of *CALM1*, *CITED1*, *FURIN*, and *TGFB3*, genes related to embryo development, were also evaluated. An effect of SP treatment in the long filamentous conceptuses for *CALM1, CITED1*, and *TGFB3* was observed. *CALM1* was the only one of these gene in which the relative expression increased in the long filamentous conceptuses recovered from mated heifers compared to control. On the other hand, both *CITED1* and *TGFB3* exhibited lower relative expression in conceptuses developing in a SP-primed environment in comparison to the control. *CALM1* encodes calmodulin 1, a calcium binding protein which represents the major calcium sensor in eukaryotes. *CALM1* has been associated to the morphogenesis process for the development of the body plan during gastrulation in response to global calcium waves (Slusarski and Pelegri, 2007), the early development of the neural system (Seto-Ohshima et al., 1987) and hematopoiesis (Kitsos et al., 2005). The participation of *TFGB3* in embryogenesis can be related to its role in the epithelial-mesenchymal transitions (EMT), which enables cell movement and morphogenesis (Zavadil and Böttinger, 2005). During gastrulation, EMT is observed in the generation of the primitive mesoderm, the cell migration into the primitive node and the establishment of the three embryonic layers (Blomberg et al., 2008; Dimitrova et al., 2017). *CITED1* (or *MSG1*), which encodes for the transcriptional factor Cbp/p300-interacting transactivator 1, has been described to be involved during embryogenesis (Dunwoodie et al., 1998; Gerstner and Landry, 2007) and placentation (Rodriguez et al., 2004; Sparrow et al., 2009) in mice. In summary, the lower expression of *CITED1* and *TGFB3*, together with higher levels of *CALM1*, suggest that long filamentous conceptus in the mated group may be at a later stage of gastrulation than morphology-matched control conceptuses. Finally, the relative expression of *IL6* and *MHC*-I (or *JSP1)*, two immune system related genes, was also evaluated, but no differences between treatments were observed.

To the best of our knowledge, this is the first study describing the effects of SP (as assessed by comparing unmated controls with heifers mated to vasectomized bulls) on the CL and early embryo development in cattle. The weight of evidence suggests that SP does not play a crucial role in embryo development in cattle as: (1) it is not clear whether SP reaches the uterus in bovine; (2) SP has been described to have a negative effect on endometrial RNA integrity *in vitro* (Fernandez-Fuertes et al., 2019); and (3) there is no evidence of an effect of SP exposure on pregnancy rates (Odhiambo et al., 2009; Pfeiffer et al., 2012; Ortiz et al., 2019). On the other hand, the embryo-related changes reported in the present work suggest that exposure to SP during natural mating changes the environment in which embryos develop from Day 7 onward. However, it is not clear whether these changes may be driven directly by the female reproductive tract or by an earlier CL maturation. Thus, further research should be conducted to elucidate the exact mechanism by which SP may improve embryo development.

## Data Availability Statement

The datasets generated for this study are available on request to the corresponding author.

## Ethics Statement

All experimental procedures involving animals were approved by the Animal Research Ethics Committee of University College Dublin, Ireland, and the Universitat de Girona, Spain, and licensed by the Health Products Regulatory Authority (HPRA), Ireland, in accordance with Statutory Instrument No. 543 of 2012 (under Directive 2010/63/EU on the Protection of Animals used for Scientific Purposes).

## Author Contributions

YM-O carried out the laboratory work, analyzed results and wrote the draft. JS, SR, SB-A, MM, and DK carried out the animal work, including handling of bulls and heifers, estrus detection, embryo transfer and recovery. MY performed statistical analysis of the data. MY, PL, JS, and BF-F contributed to the critical revision of the manuscript.

## Conflict of Interest

The authors declare that the research was conducted in the absence of any commercial or financial relationships that could be construed as a potential conflict of interest.

## Footnotes

1 https://www.ncbi.nlm.nih.gov/tools/primer-blast/
